# An automated system for polymer wear debris analysis in total disc arthroplasty using convolution neural network

**DOI:** 10.3389/fbioe.2023.1108021

**Published:** 2023-06-08

**Authors:** Sushil Kandel, Steven Su, Richard M. Hall, Joanne L. Tipper

**Affiliations:** ^1^ Faculty of Engineering and IT, University of Technology, Sydney, NSW, Australia; ^2^ College of Artificial Intelligence and Big Data for Medical Science, Shandong First Medical University & Shandong Academy of Medical Sciences, Taian, China; ^3^ School of Mechanical Engineering, University of Leeds, Leeds, United Kingdom

**Keywords:** total disc arthroplasty, UHMWPE, wear, wear particle, polymer wear debris, CNN

## Abstract

**Introduction:** Polymer wear debris is one of the major concerns in total joint replacements due to wear-induced biological reactions which can lead to osteolysis and joint failure. The wear-induced biological reactions depend on the wear volume, shape and size of the wear debris and their volumetric concentration. The study of wear particles is crucial in analysing the failure modes of the total joint replacements to ensure improved designs and materials are introduced for the next generation of devices. Existing methods of wear debris analysis follow a traditional approach of computer-aided manual identification and segmentation of wear debris which encounters problems such as significant manual effort, time consumption, low accuracy due to user errors and biases, and overall lack of insight into the wear regime.

**Methods:** This study proposes an automatic particle segmentation algorithm using adaptive thresholding followed by classification using Convolution Neural Network (CNN) to classify ultra-high molecular weight polyethylene polymer wear debris generated from total disc replacements tested in a spine simulator. A CNN takes object pixels as numeric input and uses convolution operations to create feature maps which are used to classify objects.

**Results:** Classification accuracies of up to 96.49% were achieved for the identification of wear particles. Particle characteristics such as shape, size and area were estimated to generate size and volumetric distribution graphs.

**Discussion:** The use of computer algorithms and CNN facilitates the analysis of a wider range of wear debris with complex characteristics with significantly fewer resources which results in robust size and volume distribution graphs for the estimation of the osteolytic potential of devices using functional biological activity estimates.

## 1 Introduction

Wear particles result from frictional interaction between two surfaces in motion against each other. With the ever-increasing number of complex mechanical equipment in the industrial and medical sectors, the study of wear particle characteristics and their effects on the system’s overall performance has gathered significant interest in recent years, given that wear is an inherent part of a mechanical system. Total joint replacement (TJR) procedures are performed to replace a problematic natural joint and they aim to preserve the natural range of motion. Motion preservation is achieved using an articulating mechanism which provides motion around the desired axes. These articulating components are made of various materials such as metals, polymers and ceramics. The three major joint replacements in the human body which are total hip replacement (THR), total knee replacements (TKR) and total disc replacements (TDR), use similar design principles but differ in their mechanical operation, which include different ranges of motion and different loading conditions. The difference in kinematic conditions and material choices have been shown to affect the characteristics of the wear debris produced (Milošev et al., 2012).

The study of wear debris and the potential problems it can cause in joint replacements have been carried out since the late 1960s (Lewis, 2001b). Since then, multiple studies have established cytotoxic reactions, inflammation and osteolysis as major side effects of the wear particles released from joint replacements (Harris, 1995; Ingham and Fisher, 1997; Kobayashi et al., 1997; Green et al., 2000; Lewis, 2001a; Cunningham et al., 2013). Early studies found that sub-micrometre particles were associated with macrophage responses releasing several osteolytic cytokines that cause osteolysis and aseptic loosening of the joint [13]. The level of cytokine release and the subsequent bone resorption depends on the characteristics of wear, such as particle size, shape and volumetric concentration (Green et al., 1998). Wear characteristics such as the type of wear, the rate, and its severity are the main defining characteristics of polyethylene wear. The corresponding features of wear particles that define the wear characteristics include quantity, shape, size, and composition. Various factors such as the loading conditions, polishing of the surface material, oxidative state and the level of cross-linking of polyethylene have been shown to affect the wear characteristics (Peng, 2002).

Hence, it is crucial to explore the wear characteristics to understand the biological activity of the wear particles to minimize the causes of failures, whether it be *via* a change in material choice or by the use of a different design. Numerous studies have been performed in the last 3 decades to analyse the characteristics and biological compatibility of wear debris in TJRs. Apart from the analysis of failure modes in artificial joints, wear particle analysis is one of the crucial features in the condition monitoring of industrial machines and the prediction of their imminent behaviour [6]. Unlike the online monitoring and analysis of wear debris in an industrial setup, most of the wear analysis in joint replacements is done offline by taking images of wear debris from an *in vitro* simulation. Despite similar wear modes present in joint replacements and industrial machines, the nature of wear particles is quite different in terms of the particle size and the wear volume where the particles of interest in an industrial setup are in the order of micrometers. In contrast, the particles of concern in joint replacements are in the range of nanometers to millimeters (Charnley et al., 1968).

Wear debris analysis is a time-consuming process due to the complex nature of the wear particles generated from a joint replacement. Particle sizes range from less than 50 nm to greater than 100 µm and are morphologically complex due to a lack of uniformity in size, shape and intensity (McKellop et al., 1995). Smaller particles are hard to identify due to contamination by protein structures and low resolution of imaging. Until recently, the quantitative analysis of wear debris was performed manually, adding to the time constraint. The use of computer vision and machine learning for structural and morphological analysis of the wear debris is more efficient in terms of resources and time, which allows for a larger set of data to be analysed, potentially improving the distribution functions of wear particle size and volume.

The extraction and classification of the wear debris is a major step in determining the characteristics of the wear particles. There have been a small number of attempts to classify the wear particles generated from the use of machinery in the late 1990s using neural networks (Myshkin et al., 1997; Peng and Kirk, 1998). These methods use a discrete set of values, known as features, to classify the data into different categories. The features can be any data parameter such as the area, perimeter, length, shape, Fourier descriptors and fractal dimensions. These methods can perform basic classification but fail when the dataset is complex, and the features of two datasets become similar. Classification attempts have been made using a support vector machine (SVM) classifier with dissimilarity measures as the feature descriptors to detect the progression of wear in a tribological setup with a classification accuracy of 97% for wear particle images in different stages of the simulation, without considering their morphology (Podsiadlo and Stachowiak, 2005). Their main achievement was to distinguish if a debris sample is at the initial or mid stages of wear. However, in addition to the results not being explicit about the wear regimes, the results of this study are constricted due to the use of wear debris that was uniform and carefully selected.

Eckold et al. (Eckold et al., 2015) used a scale-invariant feature transform (SIFT) to extract features from the wear particle images obtained from a total disc replacement setup, using Charite TDR implant and then used SVM to classify the wear particles based on their morphology into five different categories with an accuracy of 77.6%. Juranek et al. (Juránek et al., 2011) used a machine learning algorithm called AdaBoost as a classifier on a four-bit feature vector produced using centre-symmetric local binary patterns to classify wear particles produced in an industrial machine into four classes based on the mechanism of wear with an average classification accuracy of 91.8%.

Recent studies on the classification of wear debris produced in industrial machines have used a convolution neural network (CNN) to determine the wear phenomenon according to the type of wear debris present. In a study by Wang et al. (Wang H et al., 2019), accuracies of up to 96% were achieved in detecting the presence of wear debris with disregard to the type and quantity of wear debris. Another study used CNN to detect the wear condition present in a mechanical setup without classifying the wear debris, with an accuracy of 90% (Wang et al., 2020). However, for the detailed quantitative analysis of wear debris, the average classification accuracies for different types of wear debris ranges from 77% to 83% across multiple studies (Wang H et al., 2019; Wang et al., 2020; Wang S et al., 2019). CNN-based identification and classification of wear debris in joint arthroplasty have been shown to have very high accuracies in multi-class morphological classifications as shown in (Hu et al., 2022). However, the wear debris collected in this study was specific and constrained as they were collected from a 50 nm filter membrane Song et al. (2020) which does not accurately represent the vast number of particles generated in a joint replacement in terms of size and frequency.

Wear debris classification in the industrial setup has come a long way, with significant improvements in classification accuracy and detection of the underlying wear mechanism. However, the same can not be said for medical applications mainly because of the difference in size and number of the wear particles due to the difference in the dynamics of the two systems. The size of wear debris of interest in the industrial condition monitoring ranges from 20 µm to hundreds of micrometres (Wang H et al., 2019; Wang S et al., 2019), which can cause mechanical failure due to equipment breakages on a dynamic system. On the contrary, wear debris of a few micrometres, or less is more problematic in joint replacements as they are more biologically active and cause macrophage activation resulting in osteolysis. This difference in the size of wear particles correlates to the performance of a classifier and thus results in a significant difference in classification accuracies. Aside from that, the wear debris in the mechanical system are suspended in a lubricating medium which can be filtered, replaced, and monitored online. Whereas, the wear debris in artificial joints are in a closed loop system which cannot be monitored in real-time and replacing the lubricating medium is also not a possibility which imposes significantly more challanges in wear analysis and fault prevention. Since the biological reactions due to the wear debris depend not only on the type of wear present but also on the volume and size of the wear debris, determining just the wear mechanism is not enough for understanding adverse reactions. Hence a more complex method of wear particle identification is required for comprehensive quantitative analysis.

**FIGURE 1:**
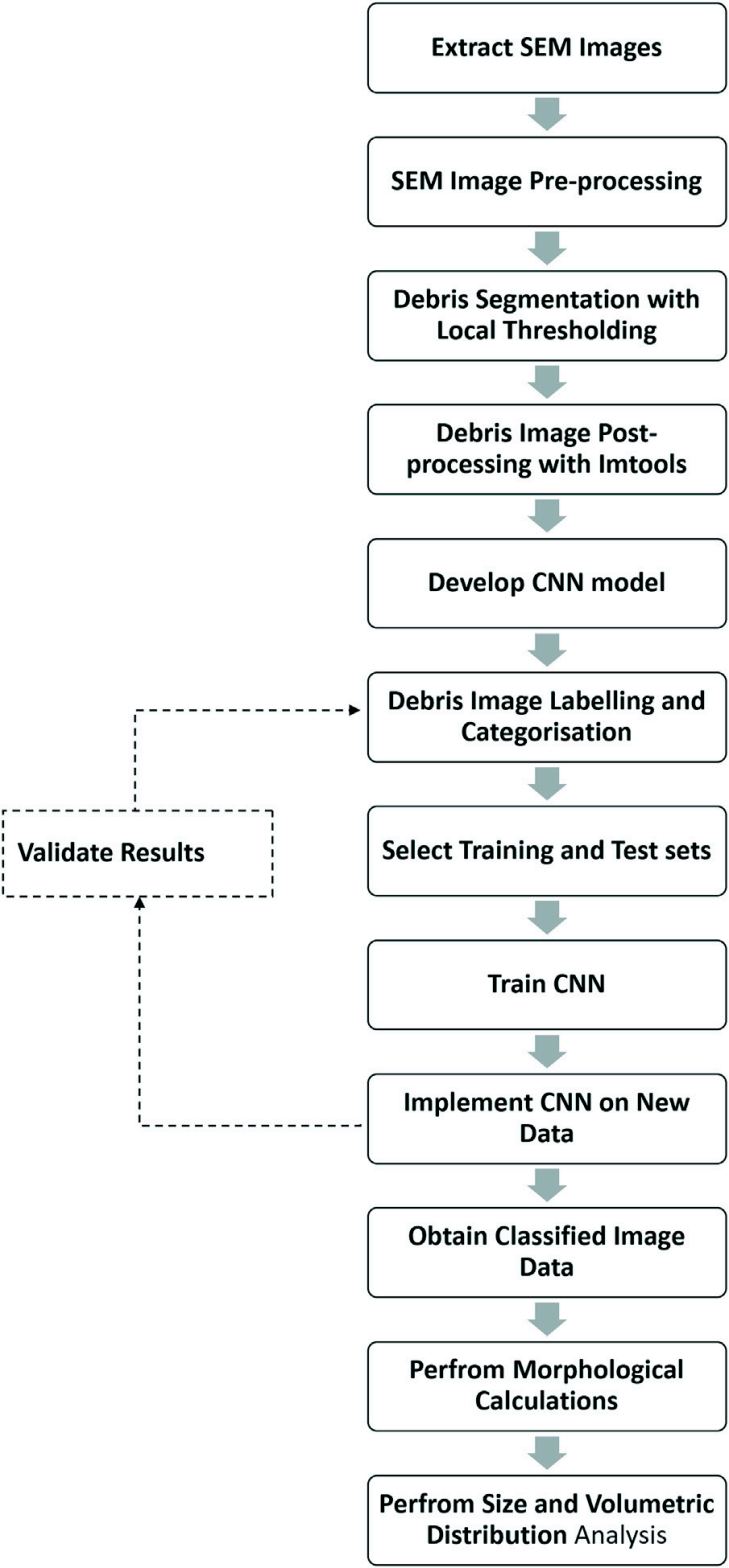
An overview of the CNN based polymer wear debris analysis system.

One of the major problems associated with wear debris analysis in joint replacements is the time required to perform image analysis and characterise wear debris, which can take up to a year for trained professionals. Another major issue is the segmentation and separation of wear debris from the non-particles present in the image. In most cases, the non-particles consist of images of filter pores. In some cases, the filter pores are well-defined and are easily identifiable. However, in other cases, the filter pores are almost identical to the wear debris and are hard to detect using traditional methods. A unique approach to tackle this issue is to use a hybrid model of wear analysis with a classical method of image segmentation and a CNN-based image classification system. To address the issues with time and the complex nature of the data, we designed a simple yet robust segmentation algorithm using adaptive thresholding to segment the image into the foreground and background. Then we used CNN to classify the objects defined by their bounding boxes into two categories of particle and non-particle. Threshold-based image segmentation is frequently used to analyse microscopic objects due to its capability to use local threshold values to identify objects in uneven illumination (Ma et al., 2023). Threshold-based segmentation is faster than machine learning-based segmentation significantly reducing the time required to perform wear debris analysis. On the other hand, a CNN is resilient to the distortion in the input image and can extract features directly from the image data, allowing better classification of particles and non-particles obtained from the SEM images. Since CNN automatically learns features from the images, the performance is significantly better, and this has been proven the case in multiple studies (Wang H et al., 2019; Wang et al., 2020; Wang S et al., 2019). With the segmentation and classification results, we then performed statistical analysis on the wear debris to perform size and volume distribution analysis. An overview of this paper is presented as a flowchart in Figure 1.

## 2 Methodology

### 2.1 Wear particle generation

Similar to Vicars et al. (Vicars et al., 2010), we performed a study to compare the results of wear of the ProDisc-L TDR from 4 degrees of freedom (DOF) and 5 DOF movement. Six ProDisc-L devices were fixed into the test cells of the Prosim 5 DOF spine simulator, and tests were carried out for 5 million cycles (MC) according to the ISO 18192-1 4DOF protocol. The lubricating solution of 25% (v/v) Bovine serum, containing the wear debris, was extracted from the test setup every third of a million cycles and stored at −20°C for analysis.

### 2.2 Debris isolation

**FIGURE 2:**
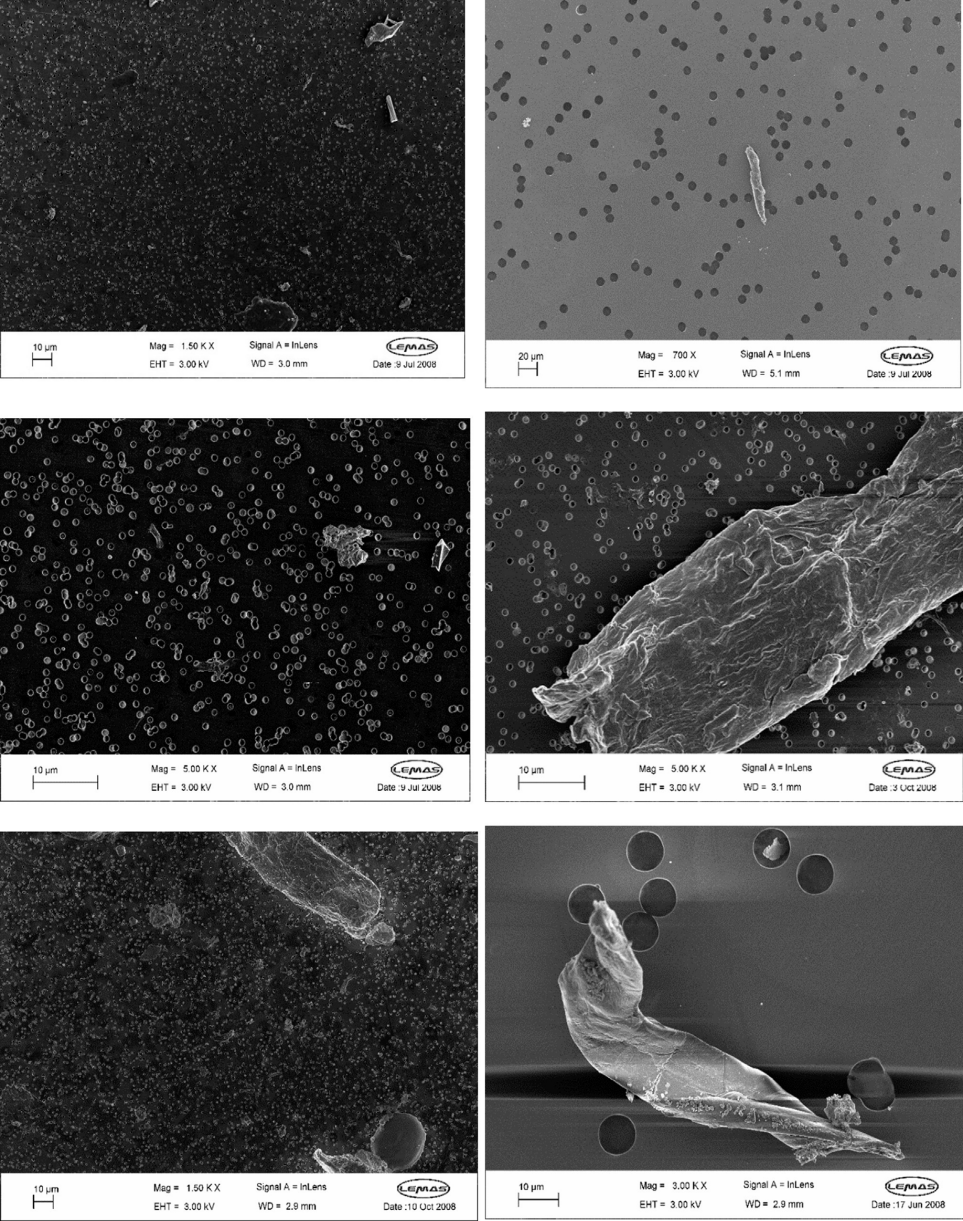
FEGSEM images of different magnification levels with different level of complexity.

Wear particles were isolated from the serum Lubricant according to Richards et al. (Richards et al., 2008). An alkaline digestion procedure was employed to isolate and collect the wear particles by successive filtration through 10 μm, 1 μm and 0.015 µm filters. Tipper et al. (Tipper et al., 2012) captured the images of each filter using high-resolution field emission gun scanning electron microscopy (FEGSEM) at different levels of magnifications ranging from ×400 to 120000 x. A total of 1233 FEGSEM images from 4 simulator stations were analysed. Figure 2 shows SEM images taken at different magnification levels containing wear debris with varying levels of complexity.

### 2.3 Debris segmentation

MATLAB (MathWorks) image processing toolbox was used to analyse the FEGSEM images. Wear particles were segmented and measured using the Regionprops MATLAB function. Local adaptive thresholding was used to separate the foreground objects from the background. Before segmentation, a Gaussian filter was used to reduce the salt and pepper noise from the image, followed by a Laplacian filter which reduces the grey levels from the image and enhances the contrast and dynamic range. The dense clusters of bright pixels in the image indicated the wear particles, and the clusters of dark pixels in circular and semi-circular shapes indicated the filter pores. The segmented objects were classified into particle and non-particle with a CNN classifier. The region properties of each bright pixel cluster and black pixel cluster were calculated, including area, diameter, perimeter, mean intensity, and centroid.

### 2.4 Performance evaluation

The performance of the custom method was compared to the results obtained from image analysis using commericially available software, ImageJ. While there are other commercially available scientific image analysis software with proprietary algorithms for debris analysis, ImageJ was selected based on the better user interface. Moreover, there have been no studies establishing significant performance differences among different image analysis software. To evaluate the performance of the segmentation and pixel measurements, uniform synthetic images with varying levels of complexity of morphological and intensity features, including triangle, circle, pentagon and dodecagon, were used. The synthetic images were segmented and measured using two comparative analysis methods. The total number of pixels in an enclosed area indicated by a single object was used as the parameter for performance evaluation, as the volume estimation is primarily based on the area of the particles. Inbuilt image processing and segmentation techniques available in ImageJ were used to segment and characterise the objects.

### 2.5 Classification

Images extracted from the SEM image were manually labelled as wear particles and non-particles which mostly included filter pores as well as some foreign particles and non-objects which were isolated due to high-intensity areas as a result of light interference. The ratio of the number of wear particles to the filter pores was significant, and thus this affected the data analysis. However, the presence of foreign objects and non-objects was relatively low and did not affect the parameter distribution. Pre-established CNN models with high levels of accuracy are available to use in the classification task, but they are more complicated and require more resources. The parameters of CNN depend on the input data type and can be tailored according to the need to design a simpler architecture that uses lower resources and less time with high accuracy.

**FIGURE 3:**
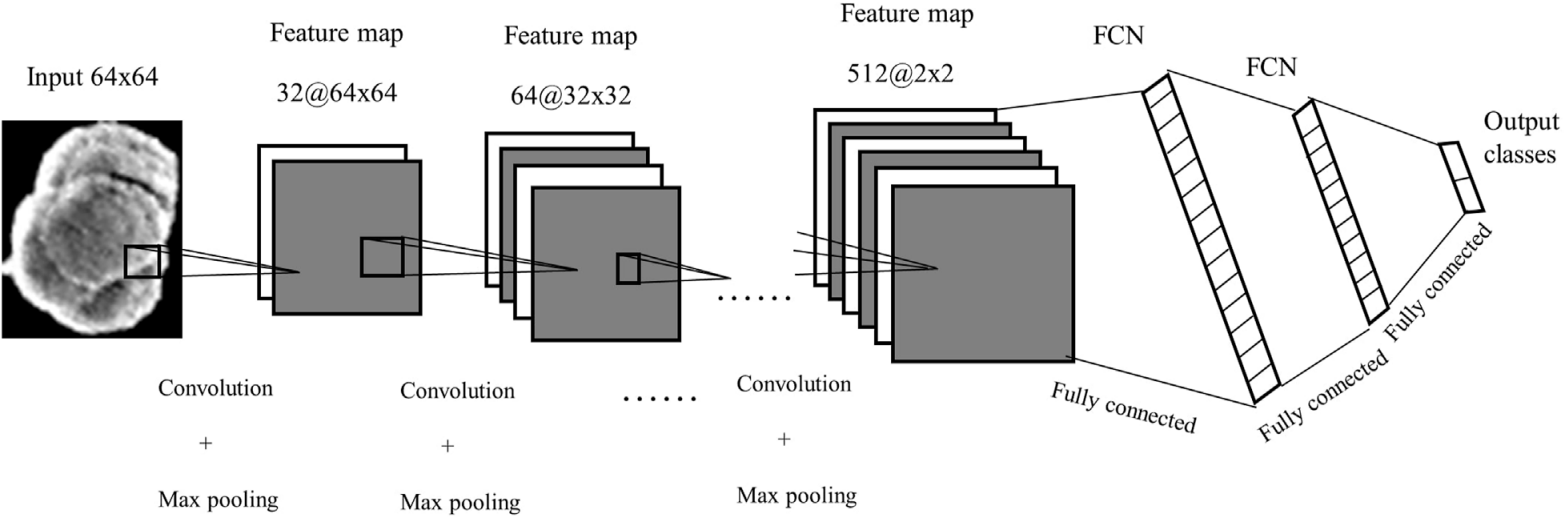
The architecture of the proposed Convolution neural network.

We designed a simple CNN structure with six convolutions and three fully connected layers. Batch normalisation and dropout techniques were used to make the CNN faster and more robust and prevent the network from overfitting. The segmented object was normalised to a size of 64 × 64 pixels and was sent as an input to the network. Six Convolution operations followed by batch normalisation, ReLu activation operations, and five max pooling operations were performed to create an output feature map of 2 × 2 × 512. Three fully connected neural networks with 2048, 1,024 and 2 neural, nodes respectively, were used to classify the information from the feature maps into two categories which indicate wear particles and non-particles. Each fully connected layer was followed by a dropout layer with a 50% probability, to avoid overfitting. A SoftMax activation function followed the fully connected layer. A simple structure of the proposed CNN is shown in Figure 3.

### 2.6 Morphological calculations

The equivalent circular diameter (ECD), Circularity (C), and elongation (E) of the particles were calculated using Eqs 1–3. The size of the particles was determined by using the length and equivalent circular diameter (ECD). The bright pixel count debotes the area of the particle and thus can be multiplied by the scale factor to convert it into micrometres square (µm^2^).

$$ECD=\sqrt{\frac{4*Area}{\pi }}$$ (1)

The circularity of the particle (C) was calculated using Eq 2:

$$C=4*Area/P^{2}$$ (2)

Elongation (E) was calculated using Eq 3:

E=Maxdia/Mindia (3)

Where, P = perimeter, MaxDia = Maximum diameter, MinDia = Minimum Diameter.

The wear particles volume was calculated by using area and wear particle thickness. Since SEM is a two-dimensional analysis, it can only provide the area of the wear particle, which is insufficient for volume analysis. Mean particle thickness can be estimated using two-dimensional area estimate and mass of the wear particles obtained by gravimetric analysis (Tipper et al., 2000). However, this method assumes a constant particle thickness which may introduce errors in volume estimations for larger particles. Another method of estimating particle thickness is using Atomic Force Microscopy (AFM) to directly measure the thickness of the particle (Wu et al., 2013). Thickness estimations for the size range < 0.1 µm, 0.1 µm–1 μm and1 µm to 10 µm were made using the width-to-thickness ratio obtained from a previous experiment that used AFM to measure the third dimension of the UHMWPE wear particles obtained from a six station pin-on-plate simulator (Wu et al., 2013). The width-to-thickness ratio used was 2.27, 1.32, 3.1 and 3.1 for the respective size ranges, which ensured a unique thickness estimation for every particle.

### 2.7 Experimental setup

Experiments conducted during this study were performed using MATLAB software on a computer with configurations: CPU i7 8,700 six cores 3.2 GHz, RAM 16 GB, GPU NVIDIA GeForce GTX 2070 8 GB. MATLAB’s built-in image processing toolbox and deep learning toolbox were used to write the segmentation and the CNN algorithms. A learning rate of 0.01, Max epochs 30, the total number of iterations 1,300, and stochastic gradient descent with momentum (sgdm) optimiser were used as training parameters. A total of 4,440 images were used in the classification, and the data was divided into 80% training and 20% test set.

## 3 Results

The custom image segmentation and analysis method was more accurate than ImageJ across all the synthetic objects, with a mean absolute percentage error of 4.04% and 14.02%, respectively. The maximum error encountered was 9.3% and 31.3%, respectively. The custom analysis method performed significantly better on objects with intensity levels close to the background intensity levels.

**FIGURE 4:**
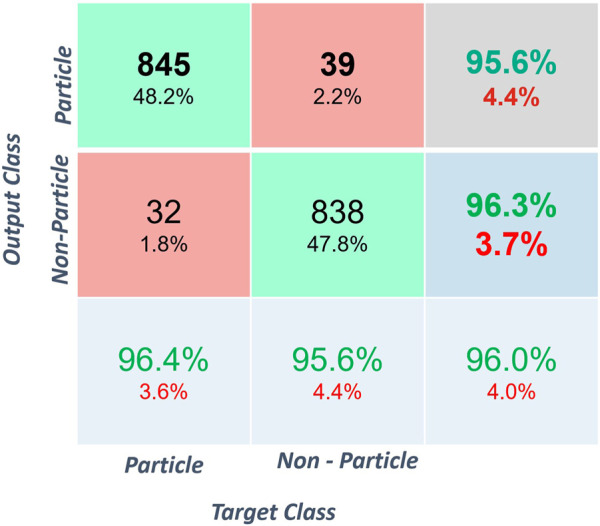
Confusion Matrix for classification.

**FIGURE 5:**
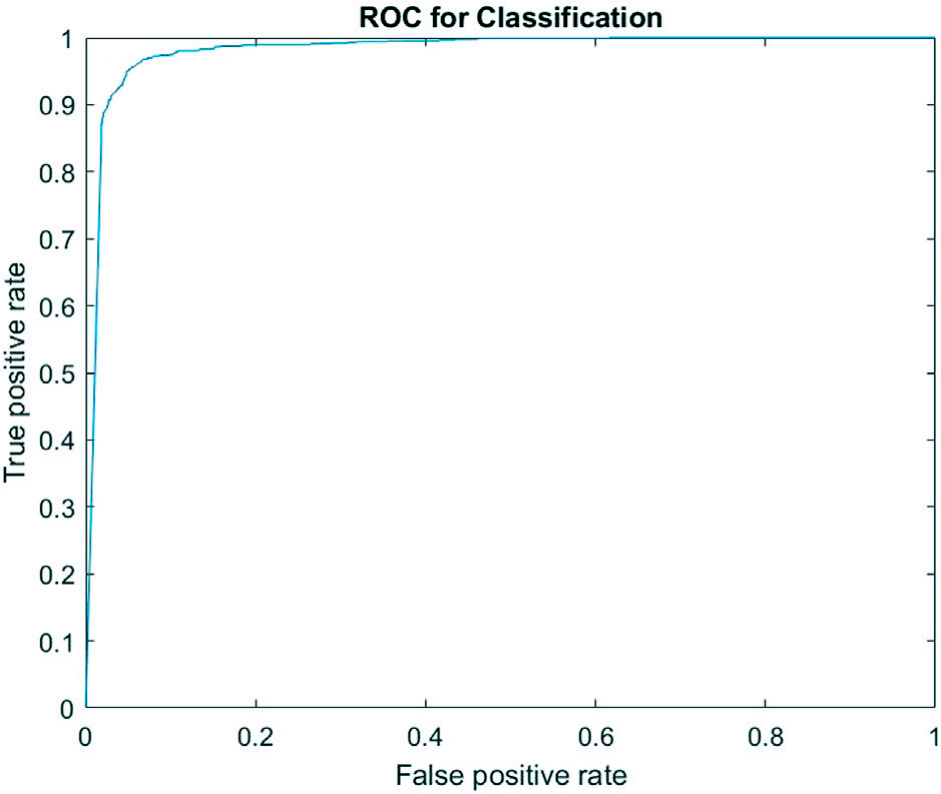
ROC curve for classification.

Four thousand four hundred objects with 2,200 particles and 2,200 non-particles were selected from a pool of labelled data for the classification. The classification results are shown in Figures 4, 5. Once the classifier was trained, it was exported to the file directory as a matrix and then used to classify newly extracted objects from the FEGSEM images for all four stations of the spine simulator. Figure 4 shows the classification confusion matrix for the respective classes. The true positive and true negative rates for both subclasses were high, suggesting an even prediction success on both classes. Figure 5 shows the ROC curve for one instance of classification with 98.17% area under the curve (AUC).

**FIGURE 6:**
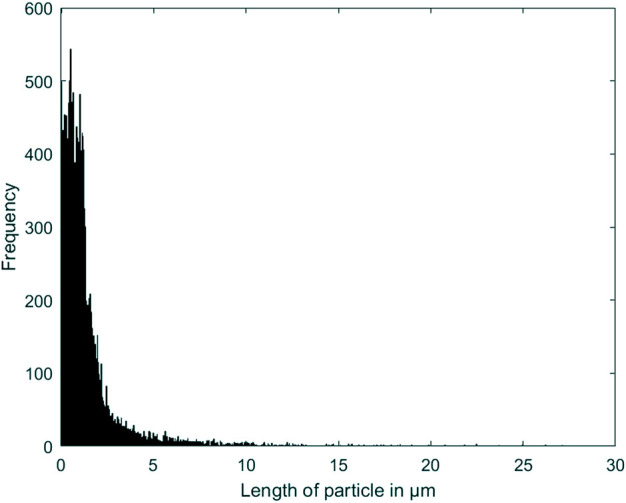
Wear particle size frequency distribution.

The CNN classifier was trained and tested on a prelabelled dataset. An accuracy of 95.61% ± 0.57% was observed, with a maximum accuracy of 96.49%. The frequency distribution of the particle size follows a log-normal distribution as shown in Figure 6. The average size of the particles was 1.699 µm with a modal size of 0.92 µm.

## 4 Discussion

This study has shown that computer vision methods can significantly improve the efficiency of wear debris analysis. The use of a convolution neural network for image classification was found to be very effective and efficient. The accuracy of the CNN classifier, as shown in Figures 4, 5, is in line with other wear-related implementations (Wang H et al., 2019; Wang et al., 2020; Wang S et al., 2019). This is a very high rate of classification accuracy, which is required to accurately measure the size and number of particles for the whole experiment. The classification accuracy was found to fluctuate between training because of the random selection of training images, which suggests that the images’ quality significantly affects the classification results. The confusion matrix, as shown in Figure 4, and ROC, as shown in Figure 5, suggest that the classifier’s performance was equal in both classes, which points to a robust classification.

**FIGURE 7:**
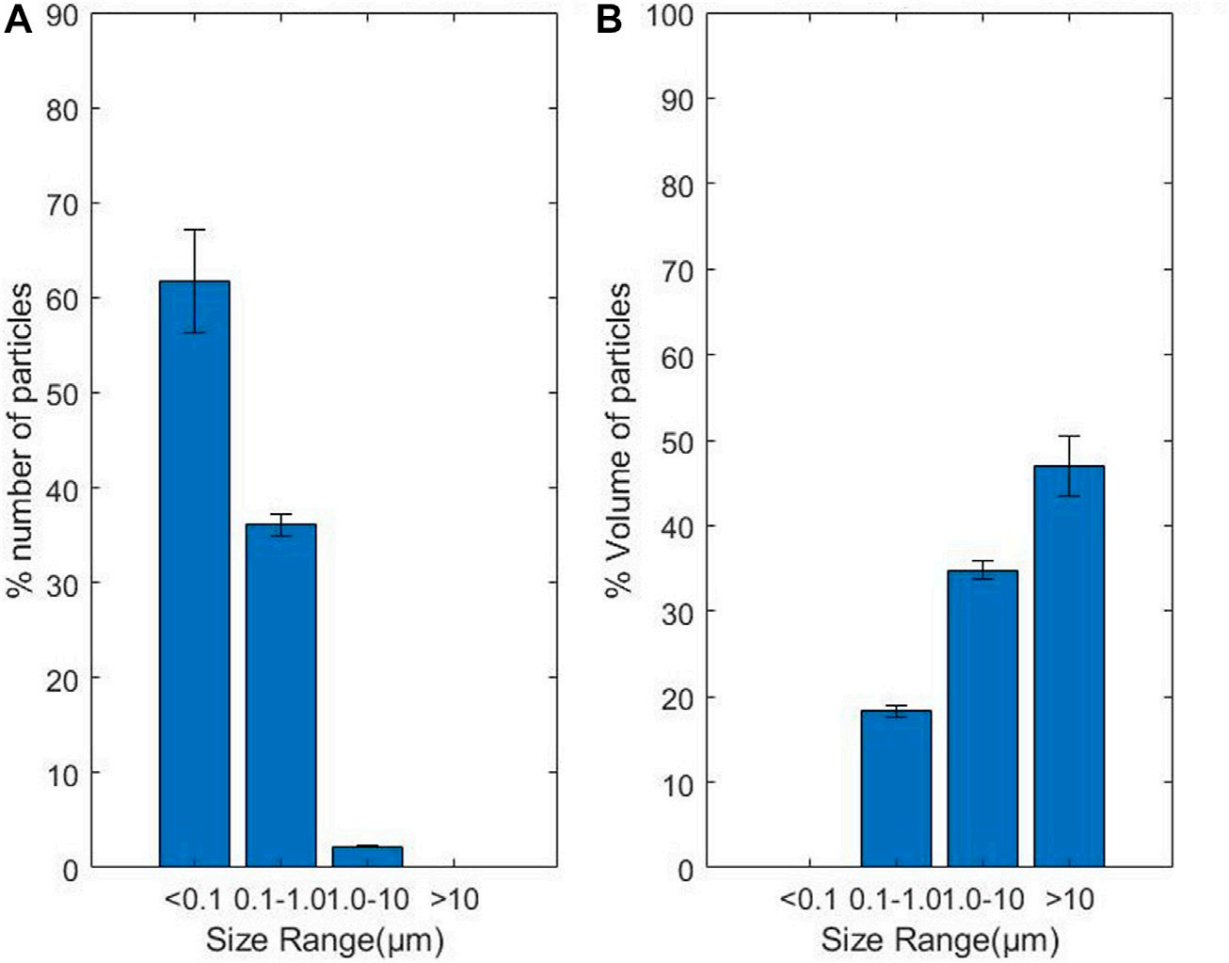
**(A)** Wear particle size distribution, **(B)** Wear particle volume distribution.

The size and volume distribution of particles, as shown in Figure 7, were similar to results from other studies (Tipper et al., 2012). Most of the particles were found to be less than 0.1 µm in length, followed by particles of size 0.1–1 μm, which are the most problematic, as established previously. Particles of size 0.1–10 µm were found to contribute more to the total area of the particles, which contradicts other similar studies (Fisher et al., 2001; Hyde et al., 2015). This difference in the area distribution may be attributed to the fact that this method relies heavily on segmentation techniques. Any error in the segmentation of large particles can a have huge effect on the area distribution. Since particles greater than 10 µm are very few, the variation in their numbers and pixel counts significantly affects the result. The area of a wear particle can vary significantly based on the accuracy of pixel detection, as demonstrated by the comparison analysis using ImageJ with uniform known shapes. The complexity of the wear particles in terms of shape and intensity levels and the intensity of the background are crucial factors to consider in improving the accuracy of the wear debris characterisation.

The accurate volume distribution is critical for analysing the biological reactivity of the implant material. The volume distribution relies heavily on the accuracy of particle area estimation and particle thickness estimation. Thickness estimation for a large number of particles is a tedious and time-consuming task. Moreover, current thickness estimation techniques, such as AFM, cannot measure the thickness of larger particles. SEM has been the standard choice for analysing large numbers of particles, as the images can be analysed later using computer-aided techniques. The accuracy of the various thickness estimation methods in past studies are debatable due to unrealistic assumptions of constant thickness across a wide range of sizes of particles obtained from different filter membranes. In this study, this problem was mitigated by using empirical thickness calculated using morphological parameters from a previous study. We have shown that the speed and accuracy of the wear particle analysis can significantly be improved by using CNN-based image analysis. However, there is still room for improvement in volume estimation.

## 5 Conclusion

Using a convolution neural network with thresholding-based segmentation can produce accurate results in segmenting and classifying wear particles with relatively low resource consumption. This segmentation and classification can be used to build a functional biological activity model for all types of wear debris under different loading conditions. However, the discrepancies in the area parameter and the lack of three-dimensional information of the particles demand improvements in the segmentation and three-dimensional particle characterisation techniques.

## Data Availability

The data analyzed in this study is subject to the following licenses/restrictions: Copyrighted material. Requests to access these datasets should be directed to JT, joanne.tipper@uts.edu.au.
